# B7-H3-mediated immune regulation in cancer: biological functions and therapeutic opportunities

**DOI:** 10.3389/fphar.2026.1953747

**Published:** 2026-09-28

**Authors:** Shreya Shreshtha, Lasini Kaluarachchige, Olivia A. Meyer, Genevieve M. Schwarz, Anil S. Basnyat, Jinyu Zhang, Yong Jiang

**Affiliations:** Department of Cell and Cancer Biology, The University of Toledo College of Medicine and Life Sciences, Toledo, OH, United States

**Keywords:** B7-H3, cancer immunotherapy, CD276, immune checkpoint, tumor immune evasion, tumor microenvironment

## Abstract

B7-H3 (CD276), a member of the B7 family of immunoregulatory ligands, has emerged as an important molecule at the intersection of tumor biology and cancer immunology. Although initially identified as a regulator of T-cell responses, accumulating evidence supports its predominantly immunosuppressive role. B7-H3 is highly expressed across diverse malignancies, including brain, prostate, lung, breast, colorectal, and pediatric cancers, while showing limited expression in most normal adult tissues. Its tumor-associated expression, including tumor vasculature, makes B7-H3 an attractive therapeutic target. Beyond immune regulation, B7-H3 promotes tumor progression through tumor cell-intrinsic mechanisms involving PI3K/AKT, NF-κB, and STAT3 signaling, angiogenesis, epithelial-to-mesenchymal plasticity, and metabolic reprogramming. Within the tumor microenvironment, B7-H3 suppresses T-cell and natural killer cell activity and promotes immunosuppressive macrophage polarization, while its expression can be regulated by metabolic and epigenetic mechanisms. These multifaceted functions have stimulated the development of diverse B7-H3-targeted therapies, including monoclonal antibodies, antibody-drug conjugates, CAR T-cell and Vδ1 T-cell therapies, nanobody-based platforms, and bispecific or trispecific immune cell engagers. Early clinical studies have demonstrated promising antitumor activity, although important challenges regarding efficacy, safety, tumor heterogeneity, and patient selection remain. In this review, we summarize the molecular regulation and biological functions of B7-H3, critically evaluate the current development of B7-H3-targeted therapies, and discuss key challenges and future directions for translating B7-H3-directed strategies into effective cancer treatments.

## 1 Introduction

The immune system relies on a sophisticated network of co-stimulatory and co-inhibitory molecules, collectively known as immune checkpoints, to regulate the magnitude and duration of T-cell responses. Therapeutic blockade of established immune checkpoints, including programmed cell death protein 1 (PD-1), programmed death-ligand 1 (PD-L1), and cytotoxic T-lymphocyte-associated protein 4 (CTLA-4), has revolutionized cancer immunotherapy. Despite these advances, a substantial proportion of patients fail to respond or eventually develop acquired resistance, highlighting the need to identify next-generation immune checkpoint targets that engage complementary immunoregulatory pathways.

Among emerging checkpoint molecules, the B7 superfamily has attracted considerable attention because of its central role in immune regulation. B7-H3 (CD276), a type I transmembrane glycoprotein first identified in 2001, has emerged as one of the most promising therapeutic targets within this family. The biological function of B7-H3 in immune regulation remains complex and context dependent. Although B7-H3 was initially characterized as a co-stimulatory molecule capable of enhancing T-cell activation, proliferation, and interferon-γ (IFN-γ) production, subsequent studies have predominantly supported a co-inhibitory role, demonstrating that B7-H3 suppresses T-cell responses and natural killer (NK) cell-mediated cytotoxicity and thereby facilitates tumor immune evasion (Guo X. et al., 2023; Flem-Karlsen et al., 2020; Getu et al., 2023; Mortezaee, 2023). These apparently contradictory findings may not necessarily represent mutually exclusive functions but could instead reflect differences in the biological and experimental contexts in which B7-H3 activity has been examined. One major unresolved issue is that the physiological receptor(s) responsible for B7-H3-mediated immune regulation has not been definitively established, raising the possibility that B7-H3 may interact with distinct receptors or signaling partners to generate stimulatory or inhibitory responses. In addition, differences in B7-H3 isoforms, species, tissue type, immune-cell composition, disease stage, and experimental systems may contribute to the divergent observations reported across studies. The tumor microenvironment may further influence B7-H3 activity through differences in cytokine composition, immune-cell infiltration, and other tissue-specific factors. Collectively, these observations suggest that B7-H3 should not be viewed as an exclusively co-stimulatory or co-inhibitory molecule; rather, its immunological function is likely determined by receptor availability and the surrounding cellular and molecular context (Zhao et al., 2022).

In addition to its immunoregulatory activity, accumulating evidence indicates that B7-H3 functions as a tumor-promoting molecule. It is highly expressed in a broad range of solid tumors and hematologic malignancies, including gliomas, prostate, lung, breast, and colorectal cancers, as well as neuroblastoma and sarcomas, whereas its expression in normal tissues is generally low and restricted (Kontos et al., 2021; Feng et al., 2021; Koumprentziotis et al., 2024). This tumor-selective expression pattern makes B7-H3 an attractive diagnostic biomarker and therapeutic target, with the potential to maximize antitumor efficacy while minimizing off-target toxicity. Increasing evidence demonstrates that B7-H3 promotes tumor cell proliferation, migration, invasion, epithelial-mesenchymal transition, angiogenesis, metabolic reprogramming, and resistance to therapy through the activation of multiple oncogenic signaling pathways (Mortezaee, 2023; Wang et al., 2020; Liu et al., 2023). The integration of these immunological and tumor-intrinsic functions establishes B7-H3 as a pivotal regulator of tumor initiation, progression, and metastasis.

Given its broad expression across human malignancies, multifaceted biological functions, and rapidly expanding therapeutic potential, B7-H3 has emerged as a compelling target for cancer immunotherapy. In this review, we summarize the current understanding of the molecular structure, regulation, and biological functions of B7-H3, with particular emphasis on its roles in tumor immunity and cancer progression. We also discuss the latest advances in B7-H3-targeted therapeutic strategies, including monoclonal antibodies, antibody-drug conjugates, bispecific antibodies, chimeric antigen receptor (CAR)-T and CAR-NK cell therapies, and radiopharmaceutical approaches. Finally, we highlight the major unresolved questions and translational challenges that must be addressed to fully realize the clinical potential of B7-H3-directed therapies.

## 2 Molecular structure and expression profile of B7-H3

### 2.1 Gene and protein structure

The B7-H3 gene (CD276) is located on human chromosome 15q23–24 and encodes a type I transmembrane glycoprotein belonging to the B7 family of immune-regulatory molecules. B7-H3 exists as two major membrane-bound isoforms that differ in the organization of their extracellular immunoglobulin-like domains (Figure 1). The 2Ig-B7-H3 isoform contains one immunoglobulin variable-like (IgV-like) and one immunoglobulin constant-like (IgC-like) domain, whereas the 4Ig-B7-H3 isoform contains a tandem duplication of these domains (IgV–IgC–IgV–IgC). In humans, 4Ig-B7-H3 is the predominant isoform, whereas 2Ig-B7-H3 is less abundant and its biological significance remains less well characterized (Guo X. et al., 2023; Getu et al., 2023; Larkin et al., 2025). Although most functional and therapeutic studies have therefore focused on 4Ig-B7-H3, whether differences in extracellular domain organization confer distinct receptor-binding properties or immunoregulatory functions remain unresolved.

**FIGURE 1:**
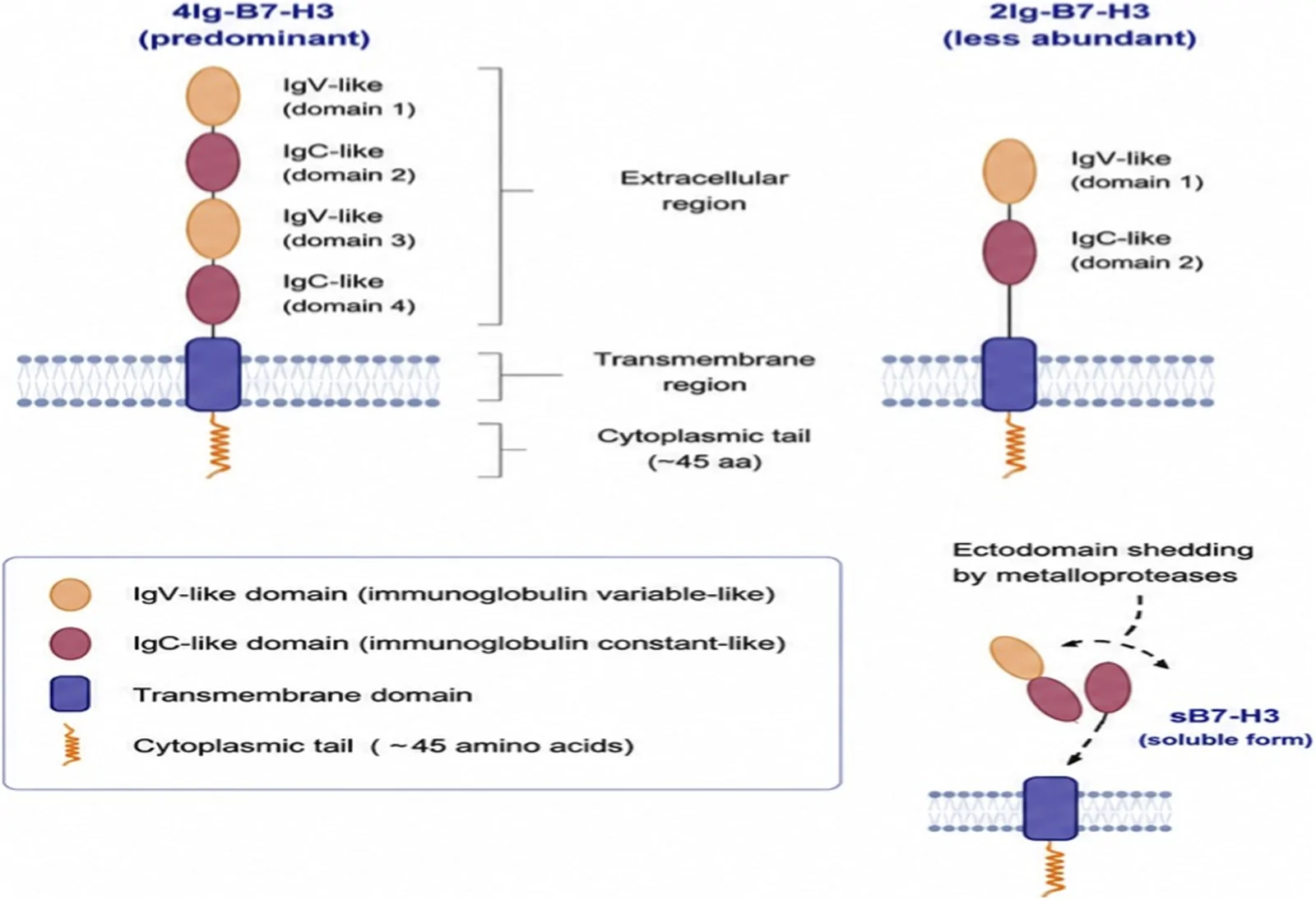
Schematic representation of the 2Ig and 4Ig B7-H3 isoforms. The schematic illustrates the structural differences between the two membrane-bound B7-H3 isoforms and the generation of soluble B7-H3.

Both isoforms possess a short cytoplasmic tail of approximately 45 amino acids. Although this intracellular region lacks canonical signaling motifs, accumulating evidence suggests that B7-H3 is capable of mediating bidirectional signaling. In addition to its role in regulating immune responses, the cytoplasmic domain has been implicated in cell-intrinsic oncogenic signaling, supporting functions that extend beyond immune modulation (Larkin et al., 2025; Alhamad et al., 2023).

Besides its membrane-bound forms, B7-H3 also exists as a soluble isoform (sB7-H3), which is generated primarily through ectodomain shedding mediated by matrix metalloproteinases. Soluble B7-H3 is detectable in serum and other body fluids, and elevated circulating levels have been associated with increased tumor burden, advanced disease, and poor clinical outcomes across a variety of malignancies. In prostate cancer, sB7-H3 has emerged as a promising liquid biopsy biomarker, with longitudinal changes in circulating levels potentially reflecting disease progression and therapeutic response (Ju et al., 2025). Nevertheless, the biological functions of sB7-H3, including whether it primarily reflects tumor-associated shedding or actively modulates immune responses, remain incompletely understood and warrant further investigation.

### 2.2 Tissue expression pattern

One of the most distinctive characteristics of B7-H3, distinguishing it from many other immune checkpoint molecules, is its markedly differential expression between normal and malignant tissues. Although B7-H3 mRNA is broadly expressed in healthy adult tissues, protein expression is generally low and restricted to a limited number of sites, including the placenta, kidney, and selected immune cell populations (Getu et al., 2023; Mortezaee, 2023; Feng et al., 2021). This discrepancy between mRNA and protein expression suggests the presence of tight post-transcriptional regulatory mechanisms controlling B7-H3 expression.

In contrast, B7-H3 protein is frequently overexpressed across a broad spectrum of human malignancies. High expression has been reported in glioblastoma and diffuse intrinsic pontine glioma (DIPG) (Vitanza et al., 2023; Nehama et al., 2019; Yüceer et al., 2025; Juković-Bihorac et al., 2025; Zhou et al., 2013; Babic et al., 2024), prostate cancer (Roth et al., 2007; Bonk et al., 2020; Benzon et al., 2017; Shi et al., 2022; Pulido et al., 2024; Shen et al., 2024), non-small-cell lung cancer (NSCLC) and small-cell lung cancer (SCLC) (Carvajal-Hausdorf et al., 2019; Jin et al., 2015; Yim et al., 2020; Fan et al., 2023; Papavassiliou et al., 2025), breast cancer, particularly triple-negative breast cancer (TNBC) (Jiang et al., 2025; Jiang et al., 2024; Kim et al., 2022; Cheng et al., 2021), colorectal cancer (Majzner et al., 2019; Gao et al., 2021; Ott et al., 2025; Hu et al., 2024; Wu et al., 2023; Chen et al., 2024; Bostanci et al., 2022; Parente et al., 2025), neuroblastoma (Rasic et al., 2026), ovarian cancer (Miyamoto et al., 2022; Zhang et al., 2017), soft tissue and bone sarcomas (He and Li, 2019; Corrigan et al., 2025; Holzmayer et al., 2024), acute myeloid leukemia (AML) (Tan and Zhao, 2024), thyroid carcinoma (Song et al., 2024), and head and neck squamous cell carcinoma (HNSCC) (Lin et al., 2021), among others (Guo X. et al., 2023; Kontos et al., 2021; Guo C. et al., 2023; Fabrizio et al., 2024; He and Li, 2019; Bottino et al., 2023; Carvajal-Hausdorf et al., 2019; Tan and Zhao, 2024; Lynch et al., 2024). The expression patterns, clinicopathological associations, prognostic significance, and corresponding levels of evidence across these malignancies are summarized in Table 1.

**TABLE 1:** B7-H3 expression profile and prognostic significance across cancer types.

Cancer type	Representative clinical cohort(s) (n)	Detection method	B7-H3 expression	Clinical association	Survival outcome	Clinical implication	Level of evidence	Key references
Glioblastoma	46; 86; 99 patients	IHC	Very high	High grade, recurrence, PD-L1 co-expression	↓ OS, ↓ PFS	Independent prognostic biomarker	Translational/Preclinical	(Nehama et al., 2019; Yüceer et al., 2025; Juković-Bihorac et al., 2025)
DIPG	Early clinical cohort	IHC	Very high	Uniform tumor expression	Preliminary clinical benefit	Excellent CAR-T target	Preclinical/Clinical	(Vitanza et al., 2023; Zhou et al., 2013)
Prostate cancer	338; >17,000 specimens	IHC	High	Gleason score, CRPC, LN metastasis	Earlier PSA recurrence	Biomarker of aggressive disease	Translational/Preclinical	(Roth et al., 2007; Bonk et al., 2020; Benzon et al., 2017; Shi et al., 2022)
NSCLC	110 patients	IHC	Moderate–High	Advanced TNM, Treg infiltration	↓ OS	Immunosuppressive phenotype	Translational/Preclinical	(Jin et al., 2015; Yim et al., 2020; Fan et al., 2023; Papavassiliou et al., 2025)
SCLC	Clinical cohorts	IHC	High	Immune-desert phenotype	Poor prognosis	Novel checkpoint target	Translational	(Carvajal-Hausdorf et al., 2019)
Breast cancer	Multiple TNBC cohorts	IHC	High	Brain metastasis, stromal expression	↓ DFS, ↓ OS	Poor prognosis	Translational/Preclinical	(Jiang et al., 2025; Jiang et al., 2024; Kim et al., 2022; Cheng et al., 2021)
Colorectal cancer	Multiple cohorts	IHC	Moderate–High	Stage, LN metastasis, angiogenesis	↓ OS, ↓ DFS	Therapeutic biomarker	Translational/Preclinical	(Majzner et al., 2019; Gao et al., 2021; Ott et al., 2025; Hu et al., 2024; Wu et al., 2023; Chen et al., 2024; Bostanci et al., 2022; Parente et al., 2025)
Neuroblastoma	81 patients	IHC	Very high	Reduced CD8 infiltration	↓ OS, ↓ EFS	High-risk biomarker	Translational/Preclinical	(Brignole et al., 2023; Majzner et al., 2019; Rasic et al., 2026; Lutskovich et al., 2025; Pulido and Nunes-Xavier, 2023)
Ovarian cancer	Clinical cohorts	IHC	High	M2 macrophage infiltration	Poor prognosis	Immunosuppressive TME	Translational/Preclinical	(Miyamoto et al., 2022; Zhang et al., 2017)
Soft tissue sarcoma	Multi-histology cohorts	IHC	High	Widespread expression	↓ PFS, ↓ OS	Pan-sarcoma target	Translational/Preclinical	(Joshi et al., 2024; Holzmayer et al., 2024)
Osteosarcoma	Clinical cohorts	IHC	High	Metastatic disease	↓ OS	Pediatric therapeutic target	Translational/Preclinical	(He and Li, 2019; Corrigan et al., 2025; Lutskovich et al., 2025)
AML	AML cohorts	Flow cytometry/IHC	Moderate	Leukemic burden	↓ OS	Emerging biomarker	Translational/Preclinical	(Tan and Zhao, 2024)
Thyroid carcinoma	Clinical specimens	IHC	Moderate	ICAM-1 co-expression	Unknown	Combination target	Translational/Preclinical	(Song et al., 2024)
HNSCC	Multi-omics cohort	RNA-seq/IHC	High	Immunotherapy resistance	Poor prognosis	Biomarker for ICI resistance	Translational	(Lin et al., 2021)

IHC, immunohistochemistry; DIPG, diffuse intrinsic pontine glioma; CRPC, castration-resistant prostate cancer; LN, lymph node; NSCLC, non-small-cell lung cancer; SCLC, small-cell lung cancer; TNBC, triple-negative breast cancer; TME, tumour microenvironment; AML, acute myeloid leukemia; HNSCC, head and neck squamous cell carcinoma; OS, overall survival; PFS, progression-free survival; DFS, disease-free survival; EFS, event-free survival; ICI, immune checkpoint inhibitor.

Immunohistochemical studies have demonstrated that B7-H3 is expressed not only on malignant cells but also within the tumor-associated vasculature. This vascular expression is of particular therapeutic interest because it provides an accessible target for antibody-based therapies and other targeted delivery strategies (Flem-Karlsen et al., 2020; Kontos et al., 2021; Larkin et al., 2025; Liu et al., 2022; Miyamoto et al., 2022). This broad distribution highlights the multifaceted role of B7-H3 in tumor progression and further supports its attractiveness as a therapeutic target (Zhou and Jin, 2021; Picarda et al., 2016).

Beyond tumor cells and the vasculature, B7-H3 is also expressed within the tumor microenvironment (TME), including tumor-infiltrating myeloid cells, cancer-associated fibroblasts, endothelial cells, and other stromal and immune cell populations (Larkin et al., 2025; Miyamoto et al., 2022; Kim et al., 2022; Cheng et al., 2021; Lin et al., 2021). These observations suggest that the biological significance of B7-H3 may depend not only on its abundance in malignant cells but also on its cellular localization and interactions with stromal and immune components of the TME.

Importantly, elevated B7-H3 expression has been associated with adverse clinicopathological characteristics and unfavorable clinical outcomes across multiple malignancies, including advanced tumor stage, metastatic progression, lymph node involvement, and reduced overall or disease-free survival (Nehama et al., 2019; Yüceer et al., 2025; Juković-Bihorac et al., 2025; Roth et al., 2007; Bonk et al., 2020; Benzon et al., 2017; Shi et al., 2022; Jin et al., 2015; Yim et al., 2020; Fan et al., 2023; Kim et al., 2022; Cheng et al., 2021; Majzner et al., 2019; Gao et al., 2021; Ott et al., 2025; Hu et al., 2024; Wu et al., 2023; Chen et al., 2024; Rasic et al., 2026; Zhang et al., 2017; Holzmayer et al., 2024; Lin et al., 2021). However, the strength of these associations varies across tumor types and is derived from different levels of evidence, ranging from preclinical and translational studies to early clinical investigations. Therefore, B7-H3 expression should be interpreted in a tumor-specific context rather than as a uniform pan-cancer prognostic marker. Collectively, these findings support the potential value of B7-H3 as both a biomarker and therapeutic target while emphasizing the need for standardized assessment of its expression, cellular distribution, and clinical significance across different malignancies.

### 2.3 Regulation of B7-H3 expression

B7-H3 expression is regulated through a complex interplay of transcriptional, signaling, and epigenetic mechanisms. Among these, the phosphoinositide 3-kinase/protein kinase B/mechanistic target of rapamycin (PI3K/AKT/mTOR) signaling pathway, a central regulator of tumor growth and survival, plays a pivotal role in controlling B7-H3 expression. Liu et al. demonstrated that activation of mTOR complex 1 (mTORC1) upregulates B7-H3 (CD276) expression on tumor cells, thereby suppressing antitumor T-cell responses and promoting immune evasion (Liu et al., 2023). Similarly, Shi et al. reported that loss of PTEN and deficiency of TP53, two of the most common molecular alterations in aggressive prostate cancer, render tumor cells highly dependent on B7-H3-mediated immunosuppression. This dependence identifies B7-H3 as a potential therapeutic vulnerability that may be exploited for the treatment of advanced prostate cancer (Shi et al., 2023).

Epigenetic regulation also plays a critical role in modulating B7-H3 expression. Ma et al. identified a previously unrecognized mechanism involving histone lactylation, a lactate-derived post-translational histone modification generated during aerobic glycolysis (the Warburg effect). Histone lactylation directly enhances CD276 transcription, thereby linking metabolic reprogramming to immune escape. These findings highlight a novel metabolic–epigenetic axis through which tumor-derived lactate promotes B7-H3-mediated immunosuppression (Ma et al., 2025). In addition, hypoxia-inducible factor-1α (HIF-1α), a master transcriptional regulator activated under hypoxic conditions, has been shown to directly induce B7-H3 expression within the tumor microenvironment, further strengthening the connection between tumor hypoxia and immune suppression (Hu et al., 2024).

Androgen receptor (AR) signaling represents an additional tumor-specific mechanism regulating B7-H3 expression. In prostate cancer, androgen stimulation has been reported to suppress B7-H3 expression, while AR-binding sites have been identified within regulatory regions of the CD276 gene, supporting direct transcriptional regulation (Benzon et al., 2017). Alterations in AR-mediated transcriptional control during progression to castration-resistant prostate cancer (CRPC) may therefore contribute to increased B7-H3 expression in advanced disease (Shi et al., 2022).

Beyond its role in cancer, B7-H3 expression can also be induced by bacterial and viral infections, suggesting that its regulation is not restricted to malignant tissues. This observation indicates that B7-H3 participates more broadly in inflammatory and innate immune responses, functioning as a versatile immunomodulatory molecule involved in both host defense and the maintenance of immune homeostasis (Tigabu, 2024). The major molecular mechanisms regulating B7-H3 expression, including their experimental evidence, human validation, and potential clinical significance, are summarized in Table 2.

**TABLE 2:** Molecular regulation of B7-H3 expression.

Regulatory pathway	Molecular mechanism	Experimental evidence	Human validation	Clinical significance	References
PI3K/AKT/mTOR	mTORC1 increases CD276 transcription	Cell lines	Yes	Immune escape	(Liu et al., 2023)
Histone lactylation	Lactate→Histone Kla→CD276	Patient tissues + mechanistic	Yes	Links metabolism with immune evasion	(Ma et al., 2025)
HIF-1α	Direct promoter activation	Human CRC	Yes	Hypoxia-induced immune suppression	(Hu et al., 2024)
PTEN/TP53 loss	Enhances B7-H3 dependence	Human CRPC	Yes	Precision biomarker	(Shi et al., 2023)
AR signalling	Direct transcriptional repression	Human prostate cancer	Yes	CRPC progression	(Benzon et al., 2017; Shi et al., 2022)
Viral/Bacterial inflammation	Inflammatory induction	Experimental	Limited	Immune regulation	(Tigabu, 2024)

## 3 B7-H3-mediated immune regulation in the tumor microenvironment

B7-H3 plays a multifaceted role in shaping the tumor immune microenvironment through coordinated effects on both adaptive and innate immune responses. Although its effects on T cells have been most extensively investigated, accumulating evidence indicates that B7-H3 also influences natural killer (NK) cells, macrophages and other myeloid populations, dendritic cells (DCs), and interactions among immune, stromal, and tumor compartments. Importantly, these immunoregulatory effects appear to be context dependent and may vary according to tumor type, cellular source and density of B7-H3 expression, and the composition of the local tumor microenvironment (TME). The major B7-H3-mediated immunosuppressive mechanisms and their potential clinical implications are summarized in Table 3 and Figure 2.

**TABLE 3:** B7-H3-mediated immunosuppressive mechanisms.

Immune cell	Mechanism	Signalling/pathway	Biological consequence	Human evidence	Clinical implication
CD8 T cells	TCR inhibition	↓IL-2, ↓IFN-γ	Reduced cytotoxicity	Strong	Immune escape
CD4 T cells	Reduced proliferation	Cytokine suppression	Poor adaptive immunity	Strong	Poor prognosis
NK cells	Inhibitory receptor signalling	Reduced degranulation	Impaired tumour killing	Strong	TriKE target
Macrophages	CCL2–CCR2	M2 polarization	Immunosuppressive TME	Strong	Combination therapy
Dendritic cells	Impaired antigen presentation	Reduced co-stimulation	Poor T-cell priming	Moderate	Vaccine resistance
Endothelial cells	VEGFA/NF-κB	Angiogenesis	Tumour progression	Strong	Anti-angiogenic target

TCR, T cell receptor.

**FIGURE 2:**
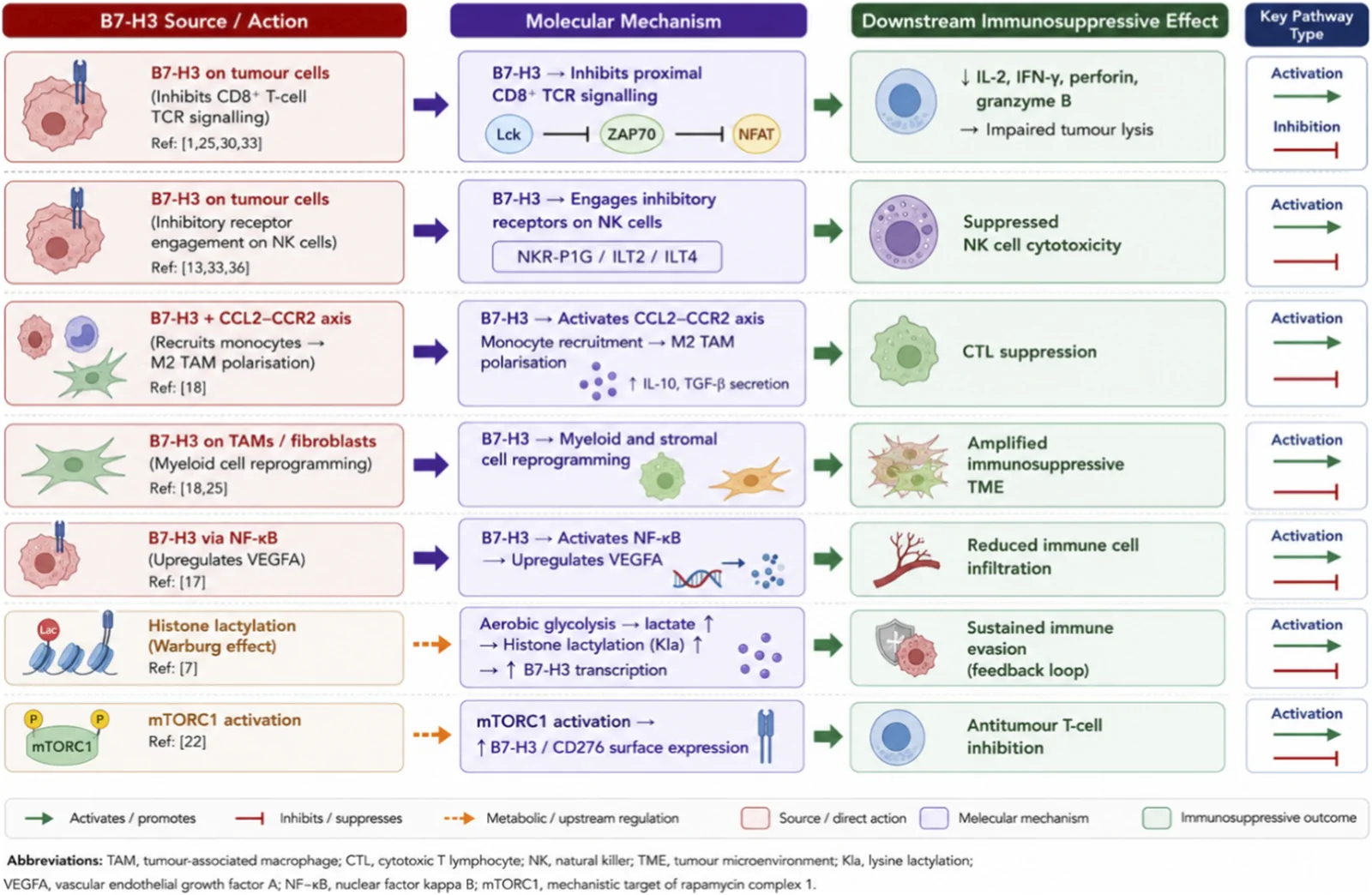
B7-H3-mediated immunosuppressive mechanisms in the tumor microenvironment. B7-H3 may regulate antitumor immunity through effects on CD8^+^ T-cell and NK-cell activity, myeloid cell recruitment and polarization, stromal cell reprogramming, and tumor-associated angiogenesis. Metabolic and upstream signaling mechanisms, including histone lactylation and mTORC1 activation, can enhance B7-H3 expression and potentially reinforce immunosuppressive signaling. Green arrows indicate activation or promotion, red blunt-ended lines indicate inhibition or suppression, and dashed orange arrows indicate upstream/metabolic regulation. TAM, tumor-associated macrophage; CTL, cytotoxic T lymphocyte; NK, natural killer; TME, tumor microenvironment; Kla, lysine lactylation; VEGFA, vascular endothelial growth factor A; NF-κB, nuclear factor kappa B; mTORC1, mechanistic target of rapamycin complex 1.

### 3.1 Regulation of T-cell responses

Accumulating evidence supports an inhibitory role for B7-H3 in adaptive antitumor immunity, although the magnitude and mechanisms of this effect appear to be context dependent. It suppresses the proliferation, cytokine production, and cytolytic activity of both CD4^+^ and CD8^+^ T cells, thereby reducing the density and functional capacity of tumor-infiltrating lymphocytes (TILs) within the tumor microenvironment (TME) (Getu et al., 2023; Mortezaee, 2023; Guo C. et al., 2023). Mechanistic studies suggest that B7-H3 may interfere with downstream T-cell receptor (TCR) signaling, resulting in reduced production of IL-2 and IFN-γ and decreased expression of cytotoxic effector molecules, although the precise receptor-dependent signaling mechanisms remain incompletely defined (Feng et al., 2021; Larkin et al., 2025; Liu et al., 2022). These effects collectively impair T-cell activation, proliferation, and antitumor cytotoxicity, thereby promoting immune escape. Despite substantial evidence supporting the immunosuppressive activity of B7-H3, its physiological receptor and the molecular basis of receptor dependent signaling remain incompletely defined. Several candidate receptors have been proposed, including triggering receptor expressed on myeloid cells-like transcript 2 (TLT-2/TREML2), interleukin-20 receptor subunit α (IL-20RA), and phospholipase A2 receptor 1 (PLA2R1); however, none has been conclusively established as the physiological B7-H3 receptor across relevant immune-cell populations. TLT-2 was initially proposed as a co-stimulatory receptor capable of enhancing CD8^+^ T-cell activation, but subsequent studies failed to reproduce a direct interaction between human B7-H3 and TLT-2, leaving this receptor–ligand relationship controversial (Guo X. et al., 2023). IL-20RA has also been reported to interact with B7-H3 and may provide a potential link to downstream signaling pathways; however, the physiological relevance of this interaction requires further validation, particularly *in vivo* (Flem-Karlsen et al., 2020). PLA2R1 has additionally been proposed as a candidate receptor, although its role in mediating the immunoregulatory effects of B7-H3 remains insufficiently characterized (Larkin et al., 2025; Corrigan et al., 2025). These unresolved and sometimes conflicting findings raise the possibility that B7-H3 may engage multiple receptors whose expression varies among immune and tumor-cell populations, potentially contributing to its context-dependent co-stimulatory and co-inhibitory activities (Getu et al., 2023). However, the molecular basis of these effects remains incompletely resolved because the physiological receptor responsible for B7-H3-mediated T-cell regulation has not been definitively identified.

Cancer-specific observations further support the relevance of B7-H3-mediated T-cell suppression. In NSCLC, B7-H3 positivity has been associated with increased FOXP3-positive regulatory T-cell infiltration and unfavorable clinical outcomes (Jin et al., 2015). In TNBC, tumor B7-H3 expression is inversely associated with CD3^+^ and CD8^+^ T-cell infiltration, while high stromal B7-H3 expression predicts shorter disease-free and overall survival (Kim et al., 2022). Similarly, elevated B7-H3 expression in neuroblastoma has been associated with reduced CD8^+^ T-cell infiltration and poorer overall and event-free survival (Rasic et al., 2026). Together, these findings indicate that the immunological consequences of B7-H3 expression are context dependent but frequently converge on impaired antitumor T-cell immunity.

### 3.2 Suppression of NK cell activity

In addition to its immunosuppressive effects on T cells, B7-H3 also impairs innate immune surveillance by inhibiting natural killer (NK) cell–mediated cytotoxicity. B7-H3 expression on tumor cells has been shown to reduce NK cell–dependent tumor lysis, potentially through engagement of inhibitory receptors or interference with the recognition of activating ligands, thereby facilitating immune evasion (Koumprentziotis et al., 2024; Bottino et al., 2023; Liu et al., 2022; Tyagi et al., 2022). This immunosuppressive mechanism has also emerged as a promising therapeutic target. Trispecific killer engager (TriKE) molecules have been developed to redirect and activate NK cells against B7-H3–expressing tumor cells, and preclinical studies have demonstrated encouraging antitumor efficacy, highlighting their potential as a novel NK cell–based immunotherapeutic strategy (Khaw et al., 2025).

### 3.3 Regulation of myeloid cell-mediated immunosuppression

Myeloid cells represent a major component of the tumor immune microenvironment and include tumor-associated macrophages (TAMs), dendritic cells (DCs), myeloid-derived suppressor cells (MDSCs), and other heterogeneous myeloid populations. Accumulating evidence suggests that B7-H3 contributes to tumor-associated immunosuppression by modulating the recruitment, differentiation, polarization, and functional states of these myeloid populations. However, the strength of evidence varies among individual cell types, with B7-H3-mediated regulation of macrophages being more extensively characterized than its effects on DCs and MDSCs. The following subsections therefore summarize the current evidence for B7-H3-mediated regulation of major myeloid cell populations.

#### 3.3.1 Tumor-associated macrophage recruitment and polarization

Emerging evidence suggests that B7-H3 may contribute to tumor-associated immunosuppression by influencing macrophage recruitment, activation, and polarization; however, these effects have been characterized primarily in specific tumor models and require broader validation across cancer types. A putative B7-H3 receptor has been identified on activated monocytes and macrophages, suggesting that B7-H3 can directly modulate myeloid cell function. In human colorectal carcinoma, B7-H3 promotes the polarization of tumor-associated macrophages (TAMs) toward an M2 phenotype and facilitates the transition from M1 to M2 macrophages during TAM differentiation (Mao et al., 2017). Similarly, Miyamoto et al. demonstrated in ovarian cancer that B7-H3 enhances TAM recruitment and M2 polarization through activation of the CCL2–CCR2 signaling axis (Miyamoto et al., 2022).

M2-polarized TAMs establish an immunosuppressive tumor microenvironment (TME) by secreting anti-inflammatory cytokines, including interleukin-10 (IL-10) and transforming growth factor-β (TGF-β), which inhibit cytotoxic T-lymphocyte (CTL) function and dampen antitumor immune responses (Xu et al., 2025). Consistent with this mechanism, inhibition of B7-H3 reduced M2 macrophage infiltration while increasing the abundance of intratumoral IFNγ^+^CD8^+^ T cells, further supporting a role for B7-H3 in orchestrating both myeloid- and lymphoid-mediated immune suppression during tumor progression (Miyamoto et al., 2022). Collectively, these findings indicate that B7-H3 drives a feedforward immunosuppressive network that promotes immune evasion and facilitates tumor progression.

This myeloid-immunosuppressive phenotype is also evident in TNBC, where B7-H3-high TAMs have been associated with enhanced tumor angiogenesis, metastatic progression, and reduced T-cell infiltration. In preclinical models, B7-H3 blockade promoted vascular normalization and enhanced the efficacy of chemotherapy and anti-PD-1 therapy (Cheng et al., 2021). These observations further support a functional link between B7-H3-positive macrophage states, immune exclusion, and tumor-promoting vascular remodeling.

#### 3.3.2 Dendritic cell regulation

B7-H3 expression on or within the dendritic-cell compartment may influence antigen presentation, co-stimulatory signaling, and subsequent T-cell priming (Mortezaee, 2023). Impaired DC function could therefore provide an additional mechanism through which B7-H3 contributes to reduced antitumor T-cell immunity. However, compared with its effects on T cells and TAMs, the molecular mechanisms and clinical significance of B7-H3-mediated DC regulation remain insufficiently characterized and require further validation.

#### 3.3.3 Myeloid-derived suppressor cells and other myeloid populations

Beyond macrophages, B7-H3 has been implicated in the regulation of other myeloid immune populations, although the available evidence is less extensive. B7-H3 has also been detected in heterogeneous immunosuppressive myeloid populations within the TME, raising the possibility that its functions extend to myeloid-derived suppressor cells and related cell states (Mortezaee, 2023). Because many of these populations exhibit substantial phenotypic plasticity, future studies incorporating single-cell and spatial approaches will be important for defining the cellular sources of B7-H3 and determining how its expression influences communication among tumor, myeloid, lymphoid, and stromal compartments.

Cancer-specific studies provide additional evidence that B7-H3 may shape heterogeneous myeloid compartments. In NSCLC, B7-H3 has been detected on tumor-associated macrophages, dendritic cells, and myeloid-derived suppressor cells, and its expression has been associated with unfavorable outcomes during PD-1 blockade (Yim et al., 2020). In AML, B7-H3 may also influence myeloid and stromal components of the bone marrow niche, although the extent of direct B7-H3-mediated regulation remains incompletely established (Lee et al., 2017; Zhang et al., 2021). These observations emphasize the need for single-cell and spatial studies to define lineage-specific B7-H3 functions across tumor contexts.

Collectively, B7-H3 contributes to tumor immune evasion through multiple interconnected mechanisms, including suppression of T-cell and NK-cell activity and modulation of immunosuppressive myeloid populations. Upstream metabolic and signaling mechanisms, including histone lactylation and mTORC1 activation, can further enhance B7-H3 expression and reinforce B7-H3-associated immunosuppression within the tumor microenvironment. These immune-regulatory interactions and their downstream consequences are summarized in Figure 2, in which activating and inhibitory relationships are distinguished using different graphical symbols and arrow styles.

## 4 Tumor-intrinsic and non-immunological functions of B7-H3

In addition to its immunoregulatory activities, B7-H3 exerts tumor-promoting functions that are independent of direct immune-cell regulation. These non-immunological effects include tumor-cell proliferation and survival, epithelial-to-mesenchymal plasticity, migration and invasion, metabolic reprogramming, therapeutic resistance, angiogenesis, and vascular remodeling. The cancer-specific evidence is integrated below according to mechanism to distinguish these tumor-intrinsic and non-immunological activities from the immune-regulatory functions discussed in Section 3.

### 4.1 Cell-intrinsic pro-tumorigenic signaling

Beyond its immunological functions, B7-H3 exerts direct cell-intrinsic oncogenic effects that contribute to tumor progression. B7-H3 expression promotes tumor cell proliferation, resistance to apoptosis, epithelial-to-mesenchymal plasticity (EMP), and enhanced migratory and invasive capacities (Mortezaee, 2023; Feng et al., 2021; Larkin et al., 2025).

Mechanistically, B7-H3 activates several major oncogenic signaling pathways, including PI3K/AKT, MAPK/ERK, STAT3, and NF-κB. Activation of these pathways is sufficient to promote tumor growth, survival, and metastatic potential, even in the absence of immune modulation (Mortezaee, 2023; Liu et al., 2022).

In addition to its role in canonical signaling pathways, Alhamad et al. identified a previously unrecognized interaction between B7-H3 and inosine-5′-monophosphate dehydrogenase 2 (IMPDH2), the rate-limiting enzyme in guanine nucleotide biosynthesis (Alhamad et al., 2023). This discovery suggests that B7-H3 also contributes to metabolic reprogramming by supporting nucleotide biosynthesis and tumor cell survival. These findings broaden the functional landscape of B7-H3 beyond immune regulation and oncogenic signaling, while highlighting a potential therapeutic vulnerability through synthetic lethality in B7-H3–overexpressing tumors.

Cancer-specific studies illustrate how these tumor-intrinsic effects are shaped by molecular context. In prostate cancer, B7-H3 expression is linked to androgen receptor (AR) signaling, and altered AR-dependent transcriptional control may contribute to increased B7-H3 expression during progression to castration-resistant disease (Benzon et al., 2017; Shi et al., 2022). PTEN loss and TP53 deficiency have also been associated with increased dependence on B7-H3, suggesting a potential basis for biomarker-guided targeting (Shi et al., 2023). In colorectal cancer, B7-H3 has been reported to enhance EGFR stability, tumor-cell proliferation, and resistance to oxaliplatin, whereas combined B7-H3 and EGFR targeting restored chemosensitivity in preclinical models (Chen et al., 2024). In ovarian cancer, B7-H3 has been associated with increased proliferation, migration, and invasion and reduced apoptosis through JAK2/STAT3 signaling (Zhang et al., 2017). In TNBC, B7-H3 blockade can induce compensatory fatty-acid synthesis, identifying adaptive metabolic reprogramming as a potential vulnerability for rational combination therapy (Jiang et al., 2025).

### 4.2 Tumor angiogenesis and vascular remodeling

B7-H3 also plays a significant role in promoting tumor angiogenesis. Wang et al. demonstrated that, in colorectal cancer, B7-H3 enhances angiogenesis by activating the NF-κB signaling pathway, thereby increasing the transcription of vascular endothelial growth factor A (VEGFA) (Wang et al., 2020).

In addition to its pro-angiogenic function, B7-H3 is constitutively expressed on tumor-associated vascular endothelial cells, suggesting a dual role in facilitating both immune evasion and vascular remodeling. This dual functionality provides a strong rationale for targeting B7-H3 therapeutically, as B7-H3-directed therapies may simultaneously enhance antitumor immunity while inhibiting tumor angiogenesis.

Furthermore, Hu et al. reported that B7-H3 promotes colorectal cancer progression by upregulating heparin-binding EGF-like growth factor (HB-EGF) through a hypoxia-inducible factor-1α (HIF-1α)-dependent mechanism (Hu et al., 2024). This finding identifies an additional non-immunological pathway by which B7-H3 contributes to tumor progression, particularly within the hypoxic tumor microenvironment.

Cancer-specific evidence further illustrates the vascular effects of B7-H3. In colorectal cancer, exosome-associated B7-H3 released by tumor cells can be internalized by endothelial cells and activate the AKT1/mTOR/VEGFA pathway, thereby enhancing endothelial migration and metastatic dissemination (Wu et al., 2023). In NSCLC, CRISPR/Cas9-based studies have implicated B7-H3 in vasculogenic mimicry, a tumor-cell-derived vascular-like program that may contribute to aggressive behavior (Fan et al., 2023). In TNBC, B7-H3-high TAMs are associated with abnormal angiogenesis, whereas B7-H3 blockade promotes vascular normalization in preclinical models (Cheng et al., 2021). Together, these findings indicate that B7-H3 can influence tumor vascular biology through both tumor-cell-intrinsic signaling and communication with non-immune components of the microenvironment.

Collectively, B7-H3 contributes to cancer progression through tumor-cell-intrinsic and non-immunological mechanisms that complement its immunoregulatory activities. By activating oncogenic signaling pathways, supporting metabolic adaptation, and promoting tumor angiogenesis and vascular remodeling, B7-H3 can enhance tumor growth, survival, invasion, and metastatic progression independently of direct immune-cell regulation. These multifaceted functions broaden the biological significance of B7-H3 beyond immune checkpoint regulation and provide additional mechanistic rationales for B7-H3-targeted therapeutic strategies.

## 5 B7-H3-targeted therapeutic approaches, clinical translation, and future perspectives

The favorable tumor-associated expression profile of B7-H3 has supported the development of diverse therapeutic platforms, including Fc-engineered monoclonal antibodies, antibody–drug conjugates (ADCs), bispecific targeting strategies, chimeric antigen receptor (CAR) T-cell therapies, and NK-cell-engaging approaches. These modalities differ substantially in their mechanisms of action, developmental stages, antitumor efficacy, and safety profiles. A comparative overview of representative B7-H3-targeted therapeutic modalities and their current preclinical and clinical development is summarized in Table 4. This unified section also considers clinical translation, therapeutic resistance, biomarker-guided patient selection, and future directions to provide a cohesive view of the development and optimization of B7-H3-directed therapies (Smith et al., 2026; Guo et al., 2025).

**TABLE 4:** Summary of B7-H3-targeted therapeutic modalities.

Modality	Agent	Cancer type	Patients (n)	Major efficacy	Survival/clinical outcome	Stage	Major toxicity	Referemces
mAb (Fc-engineered)	Enoblituzumab (MGA271)	Solid tumours	∼179	Durable responses	Clinical activity	Phase I/II	Favourable safety; anti-tumour activity	(Kontos et al., 2021; Feng et al., 2021; Koumprentziotis et al., 2024)
ADC (Topo I inh.)	DS-7300a (ifinatamab deruxtecan)	SCLC	21 (SCLC subset)	ORR 52.4%	mPFS 5.6 mon; mOS 12.2 mon	Phase I/II	Tumour responses in pretreated pts	(Feng et al., 2021; Wang et al., 2020)
ADC (DNA alkylator)	Vobramitamab duocarmazine	Neuroblastoma	Ongoing	Promising	Early evaluation	Phase I	Potent preclinical; early clinical evaluation	(Liu et al., 2023)
Bispecific ADC	Anti-PD-L1 and B7-H3 ADC	Solid tumours	N/A	N/A	N/A	Preclinical	Superior efficacy vs. monospecific; immune activation	(Larkin et al., 2025)
CAR T (αβ)	B7-H3 CAR T cells	DIPG, GBM	23 enrolled/21 treated	Radiographic responses	CNS immune activation	Phase I	First-in-human: biological activity, safe	(Alhamad et al., 2023; Ju et al., 2025; Guo C. et al., 2023; Fabrizio et al., 2024)
CAR T (co-targeting)	B7-H3 + CSPG4 CAR T	TNBC	N/A	Better tumour coverage	N/A	Preclinical	Superior coverage vs. single-antigen	(He and Li, 2019)
CAR Vδ1 T cells	B7-H3 CAR-Vδ1T	Multiple solid tumours	N/A	N/A	N/A	Preclinical	Broad-spectrum anti-tumour activity	(Bottino et al., 2023)
TriKE (NK engager)	B7-H3 × CD16 × IL-15 TriKE	Head and neck cancer	N/A	Strong NK activation	N/A	Preclinical	Potent NK cell-mediated cytotoxicity	(Carvajal-Hausdorf et al., 2019)

### 5.1 Monoclonal antibodies

Monoclonal antibodies (mAbs) represent the most advanced class of B7-H3-targeted therapeutics. Among these agents, Enoblituzumab (MGA271) is the most extensively characterized. It is a humanized, Fc-engineered IgG1 monoclonal antibody designed to enhance antibody-dependent cellular cytotoxicity (ADCC). Comprehensive reviews by Kontos et al. and Michelakos et al. summarize the current landscape of B7-H3-targeted antibodies, highlighting the diverse antibody formats under development and their underlying mechanisms of action (Kontos et al., 2021; Michelakos et al., 2021; Feng et al., 2021; Loo et al., 2012). A major advantage of B7-H3-targeted mAbs is the broad expression of B7-H3 on both tumor cells and the tumor-associated vasculature. This dual expression enhances antibody accessibility to the tumor microenvironment and enables ADCC-mediated elimination of both malignant cells and tumor-supporting endothelial cells, thereby potentially improving antitumor efficacy (Kontos et al., 2021; Feng et al., 2021; Koumprentziotis et al., 2024).

### 5.2 Antibody–drug conjugates (ADCs)

Antibody–drug conjugates (ADCs) have emerged as one of the most promising therapeutic strategies for targeting B7-H3. These agents consist of a tumor-specific monoclonal antibody linked to a highly potent cytotoxic payload through a chemical linker, enabling the selective delivery of cytotoxic agents to B7-H3–expressing tumor cells while minimizing systemic exposure and off-target toxicity.

Several B7-H3-targeted ADCs are currently under preclinical and clinical development. DS-7300a (ifinatamab deruxtecan), which carries a DNA topoisomerase I inhibitor payload, has demonstrated robust antitumor activity and pronounced bystander killing effects across multiple preclinical tumor models (Liu et al., 2022). Similarly, vobramitamab duocarmazine, an ADC incorporating a duocarmycin-based DNA-alkylating payload, has shown potent antitumor efficacy in preclinical neuroblastoma models and has subsequently advanced into clinical evaluation (Brignole et al., 2023).

Beyond conventional ADCs, novel dual-targeting approaches are also being explored. Dong et al. developed a bispecific ADC simultaneously targeting PD-L1 and B7-H3, which exhibited superior antitumor activity compared with single-target strategies. In addition to its direct cytotoxic effects, this bispecific ADC enhanced antitumor immunity by promoting the infiltration and activation of tumor-infiltrating lymphocytes (TILs), suggesting a synergistic mechanism involving both targeted drug delivery and immune modulation (Dong et al., 2024).

Furthermore, Feustel et al. comprehensively reviewed the current landscape of B7-H3-targeted therapeutics, including ADC-based approaches, and summarized early clinical evidence demonstrating encouraging safety profiles and preliminary antitumor efficacy, supporting the continued clinical development of B7-H3-directed ADCs (Feustel et al., 2024).

### 5.3 CAR T-cell therapies

Chimeric antigen receptor (CAR) T-cell therapies targeting B7-H3 have rapidly advanced from preclinical validation to early-phase clinical evaluation, driven by the broad expression of B7-H3 across multiple malignancies and its limited distribution in normal tissues (Du et al., 2019; Zhang et al., 2020; Lichtman et al., 2021). This expression profile makes B7-H3 an attractive target for CAR T-cell–based immunotherapy.

Pioneering work by Majzner et al. demonstrated that B7-H3-targeted CAR T cells exert potent cytotoxic activity against a broad spectrum of pediatric solid tumors and central nervous system malignancies in both *in vitro* and *in vivo* models (Majzner et al., 2019). These findings were subsequently reinforced by Inthanachai et al., who developed optimized B7-H3 CAR constructs with enhanced antitumor efficacy in glioblastoma models (Inthanachai et al., 2025). Building on these preclinical successes, clinical translation has begun. In the first-in-human study by Vitanza et al., intraventricular administration of B7-H3-targeted CAR T cells in patients with diffuse intrinsic pontine glioma (DIPG) demonstrated encouraging preliminary evidence of biological activity, including radiographic tumor responses and signs of central nervous system immune activation (Vitanza et al., 2023).

To further enhance therapeutic performance, several innovative CAR designs have been investigated. Li et al. developed nanobody-based B7-H3 CAR T cells incorporating camelid-derived single-domain antibodies, which offer potential advantages such as a smaller molecular size, lower immunogenicity, improved tumor penetration, and enhanced manufacturing flexibility (Li D. et al., 2023). In a separate study, the same group also demonstrated robust antitumor activity of B7-H3-targeted CAR T cells in preclinical prostate cancer models, supporting the broader applicability of this therapeutic strategy beyond pediatric and brain tumors (Bottino et al., 2023; Li S. et al., 2023).

Strategies to overcome tumor antigen heterogeneity have also shown promising results. Stucchi et al. engineered dual-targeting CAR T cells directed against both B7-H3 and CSPG4, achieving superior antitumor efficacy compared with single-antigen CAR T cells in triple-negative breast cancer (TNBC) models (Stucchi et al., 2025). Expanding the repertoire of cellular immunotherapies, Jiang et al. demonstrated the feasibility of B7-H3-targeted CAR γδ T cells, highlighting an alternative immune effector platform that may offer distinct biological and therapeutic advantages over conventional αβ CAR T cells (Alhamad et al., 2023; Ju et al., 2025; Guo C. et al., 2023; Fabrizio et al., 2024). Collectively, these studies underscore the rapid evolution of B7-H3-directed CAR T-cell therapy and support its continued development as a promising treatment strategy for a wide range of solid tumors.

### 5.4 Bispecific and trispecific engagers

Bispecific and trispecific engager molecules represent an emerging class of B7-H3-targeted immunotherapies that redirect immune effector cells to tumor sites without requiring patient-specific cell manufacturing. By simultaneously engaging immune cells and tumor-associated antigens, these multifunctional agents enhance antitumor immune responses while offering the advantages of off-the-shelf therapeutic platforms.

Khaw et al. developed a trispecific killer engager (TriKE) that simultaneously binds CD16 on natural killer (NK) cells, delivers IL-15-mediated co-stimulation to promote NK cell activation and persistence, and targets B7-H3 on tumor cells (Wang et al., 2020; Carvajal-Hausdorf et al., 2019). In preclinical head and neck cancer models, this strategy elicited potent NK cell–mediated cytotoxicity and demonstrated enhanced antitumor activity (Khaw et al., 2025).

Expanding on the concept of multispecific targeting, Dong et al. developed a bispecific PD-L1/B7-H3 antibody–drug conjugate (ADC) that combines dual immune checkpoint blockade with targeted cytotoxic payload delivery (Dong et al., 2024). By simultaneously inhibiting PD-L1- and B7-H3-mediated immune suppression while directly inducing tumor cell death, this approach achieved both immune reinvigoration and enhanced antitumor efficacy, highlighting the therapeutic potential of multifunctional B7-H3-targeted immunotherapies (Dong et al., 2024).

### 5.5 Combination strategies

Given that immune checkpoint inhibition alone often fails to produce durable responses in solid tumors, combination strategies incorporating B7-H3–targeted therapies are being actively investigated to enhance therapeutic efficacy. These approaches are designed to simultaneously target complementary oncogenic and immunosuppressive pathways. For example, the bispecific PD-L1/B7-H3 antibody–drug conjugate (ADC) enables concurrent blockade of the PD-1/PD-L1 axis and B7-H3–mediated immune suppression, thereby enhancing anti-tumor activity (Dong et al., 2024).

Metabolic reprogramming also influences the response to B7-H3–targeted therapy. Jiang et al. demonstrated that B7-H3 inhibition induces compensatory upregulation of fatty acid synthesis, creating a metabolic vulnerability that can be therapeutically exploited. Combining B7-H3 blockade with fatty acid synthesis inhibitors produced synergistic anti-tumor effects, highlighting the potential of metabolism-based combination strategies (Jiang et al., 2025).

Despite these advances, several mechanisms may limit the efficacy of B7-H3–targeted monotherapy. Zhao et al. and Corrigan et al. identified antigen downregulation, intratumoral heterogeneity, and the persistence of an immunosuppressive tumor microenvironment (TME) as major barriers to sustained therapeutic responses (He and Li, 2019; Zhao et al., 2025). These findings provide a strong rationale for combinatorial approaches, particularly those that simultaneously modulate the TME. Furthermore, the frequent co-expression of B7-H3 with other immunosuppressive molecules on tumor vasculature and tumor-associated macrophages supports the development of multi-targeted therapeutic strategies to overcome resistance and improve the durability of anti-tumor responses.

### 5.6 Challenges, resistance mechanisms, and future perspectives

Despite the encouraging preclinical and early clinical activity of B7-H3-directed therapies, several biological and translational challenges may limit their efficacy. A major concern is heterogeneous B7-H3 expression, both among patients and within individual tumors. Although B7-H3 is frequently enriched in malignant tissues, its expression can vary across tumor regions, cellular compartments, disease stages, and metastatic sites. Such heterogeneity may allow B7-H3-low or B7-H3-negative tumor populations to escape antigen-directed therapies and contribute to disease recurrence. This issue may be particularly relevant to CAR T-cell therapies and antibody–drug conjugates (ADCs), whose efficacy depends on sufficient and accessible surface antigen expression. Although persistent B7-H3 expression has been observed in some settings, including recurrent GBM and sequential biopsies from metastatic colorectal cancer, these observations may not be generalizable across malignancies. Therefore, longitudinal and spatial assessment of B7-H3 expression will be important for defining the stability of the target during tumor evolution and treatment.

#### 5.6.1 Resistance mechanisms

Therapeutic resistance may arise through both antigen-dependent and antigen-independent mechanisms. Antigen loss, downregulation, or selection of pre-existing B7-H3-low tumor-cell populations may reduce the effectiveness of antigen-directed therapies. In addition, adaptive changes within the tumor microenvironment, including recruitment of immunosuppressive myeloid populations, T-cell dysfunction or exhaustion, and compensatory activation of alternative immune checkpoints, may limit the durability of B7-H3-directed immunotherapies. For cellular therapies, inadequate trafficking, limited tumor infiltration, and reduced T-cell persistence represent additional barriers, particularly in solid tumors and CNS malignancies. Preclinical strategies such as CXCR3-A engineering to improve CAR T-cell trafficking and combination approaches designed to increase tumor susceptibility illustrate potential approaches to overcoming these limitations; however, their clinical benefit remains to be established.

#### 5.6.2 Biomarker-guided patient selection

Another major challenge is the development of predictive biomarkers for patient selection. B7-H3 expression alone may not be sufficient to predict therapeutic response, particularly because different therapeutic platforms have distinct requirements for target density, cellular localization, and accessibility. Standardized and quantitative assessment of B7-H3 expression will therefore be necessary to define clinically meaningful thresholds for individual therapeutic modalities. In addition to total expression, the spatial distribution of B7-H3 between tumor, stromal, vascular, and immune compartments may influence therapeutic activity. Tumor-specific molecular features may provide additional predictive information; for example, PTEN loss and TP53 deficiency have been associated with enhanced sensitivity to B7-H3 targeting in prostate cancer, whereas combined assessment of B7-H3 and immune-cell infiltration has shown prognostic relevance in neuroblastoma. Future clinical trials should therefore incorporate integrated biomarker strategies combining B7-H3 expression and antigen density with tumor genotype, immune contexture, and other disease-specific characteristics.

#### 5.6.3 Translational challenges

Several additional challenges must be addressed during clinical translation. First, the relatively restricted expression of B7-H3 in normal tissues provides an attractive therapeutic window, but low-level expression in normal cells and vasculature raises the possibility of on-target/off-tumor toxicity, particularly with highly potent modalities such as CAR T cells, ADCs, and radiolabeled antibodies. Second, conventional xenograft and immunodeficient mouse models may not fully reproduce the complexity of the human immune microenvironment, antigen distribution, or treatment-related toxicities, potentially contributing to discrepancies between preclinical efficacy and clinical outcomes. Third, optimal route of administration, dosing, treatment sequence, and combination strategies are likely to differ among tumor types and therapeutic platforms. Locoregional delivery, as explored for B7-H3 CAR T cells in CNS malignancies, may improve tumor exposure in anatomically restricted disease, whereas systemic approaches may be more appropriate for disseminated malignancies. In addition, next-generation strategies integrating multi-antigen CAR design, nanomedicine-enabled delivery, metabolic reprogramming, and AI-assisted optimization may provide new opportunities to overcome antigen heterogeneity, impaired trafficking, and T-cell exhaustion in B7-H3-positive solid tumors.

#### 5.6.4 Future directions

Overcoming these limitations will likely require a shift from broadly applying B7-H3-targeted therapies to tumor- and biomarker-informed treatment strategies. Multi-antigen or logic-gated cellular therapies may reduce antigen-escape mechanisms, while improved CAR designs may enhance trafficking, persistence, and resistance to T-cell exhaustion. Rational combinations with immune checkpoint inhibitors, chemotherapy, targeted agents, anti-angiogenic strategies, or metabolic interventions may address compensatory resistance mechanisms, although such combinations should be guided by tumor-specific biology rather than applied uniformly across malignancies. Importantly, prospective clinical trials incorporating standardized B7-H3 assessment, longitudinal tumor sampling, pharmacodynamic biomarkers, and systematic evaluation of resistance mechanisms will be essential for determining which patients benefit from each therapeutic modality. Ultimately, successful clinical translation of B7-H3-directed therapy will depend not only on the presence of the target but also on understanding antigen density and heterogeneity, cellular localization, tumor molecular context, immune microenvironment, and mechanisms of therapeutic resistance.

## 6 Clinical trials and translational landscape

The clinical translation of B7-H3–targeted therapies has accelerated markedly in recent years, with an expanding number of Phase I and II clinical trials evaluating multiple therapeutic platforms across a broad spectrum of solid and hematologic malignancies. A comprehensive overview of the current clinical landscape has been provided by Feustel et al., who systematically categorize ongoing studies according to therapeutic modality, tumor type, and stage of clinical development (Feustel et al., 2024).

Among antibody-based strategies, enoblituzumab (MGA271) remains the most extensively investigated B7-H3–targeted monoclonal antibody. Early-phase clinical studies have demonstrated a favorable safety profile together with preliminary evidence of antitumor activity in patients with melanoma, prostate cancer, and other advanced solid tumors. Although early combination studies with PD-1 inhibitors yielded mixed results and did not consistently demonstrate synergistic efficacy, accumulating evidence suggests that appropriately designed combination regimens, including optimized patient selection and treatment sequencing, may yet enhance therapeutic benefit.

Antibody–drug conjugates (ADCs) targeting B7-H3 have also emerged as a promising therapeutic class. DS-7300a (ifinatamab deruxtecan), a DXd-based ADC developed by Daiichi Sankyo, is currently undergoing clinical evaluation in small-cell lung cancer (SCLC) and several other B7-H3–expressing malignancies. Preliminary clinical findings have demonstrated encouraging objective response rates and durable antitumor activity in heavily pretreated patients, supporting further clinical development. Recent updates on the clinical performance of this agent have been comprehensively summarized by Fabrizio et al. (2024), Feustel et al. (2024).

Cell-based immunotherapies have likewise progressed into clinical evaluation. A landmark first-in-human study by Vitanza et al. investigated the intraventricular administration of B7-H3–targeted chimeric antigen receptor (CAR) T cells in patients with diffuse intrinsic pontine glioma (DIPG) (Song et al., 2025; Timpanaro et al., 2026). The study demonstrated early signs of biological activity, including radiographic tumor responses and evidence of central nervous system immune activation, while maintaining an acceptable safety profile (Vitanza et al., 2023). These findings represent an important milestone in the clinical development of B7-H3-directed cellular therapies and provide a strong rationale for ongoing dose-escalation and efficacy studies in both pediatric and adult central nervous system malignancies.

Despite these encouraging advances, the majority of B7-H3–targeted therapies remain in the early stages of clinical development, with most ongoing studies still in Phase I. Current clinical efforts are primarily focused on malignancies characterized by high B7-H3 expression, including prostate cancer, small-cell lung cancer, brain tumors, neuroblastoma, and breast cancer. The current clinical and translational landscape of B7-H3-targeted therapies, including major therapeutic platforms, representative agents, tumor indications, and stages of clinical development, is summarized in Table 5.

**TABLE 5:** Key clinical studies of B7-H3-targeted therapies.

Agent	Trial	Phase	Tumour	Patients (n)	ORR	Survival	Safety	Clinical significance
Enoblituzumab (MGA271)	NCT01391143	I	Advanced solid tumours	179	Clinical responses observed	Durable disease control	No DLT; low grade ≥3 AEs	First proof-of-concept mAb
DS-7300a	Phase I	I/II	SCLC	21	52.4%	mPFS 5.6 months; mOS 12.2 months	Manageable	Most promising ADC
Vobramitamab duocarmazine	Ongoing	I	Neuroblastoma	—	Pending	Pending	Acceptable	Pediatric ADC
B7-H3 CAR-T	BrainChild-03	I	DIPG	23 enrolled/21 treated	Radiographic responses	Early clinical benefit	Well tolerated	First CNS CAR-T
Prostate CAR-T	Preclinical	—	CRPC	—	—	—	—	Supports clinical translation

A notable trend in the translational landscape is the increasing incorporation of biomarker-driven trial designs aimed at improving patient selection and maximizing therapeutic efficacy. Biomarkers such as B7-H3 expression assessed by immunohistochemistry, together with emerging liquid biopsy–based assays, are being integrated into clinical protocols to identify patients most likely to benefit from B7-H3-targeted therapies. This evolution toward precision oncology is expected to enhance clinical outcomes and facilitate the successful development of next-generation B7-H3-directed immunotherapies (Ju et al., 2025).

## 7 Discussion

The evidence summarized in this review establishes B7-H3 as a multifunctional oncologic target with broad therapeutic relevance. Its widespread expression across both solid and hematologic malignancies, coupled with relatively limited expression in normal tissues, provides a favorable therapeutic index that compares favorably with many established immunotherapy targets. Beyond serving as a tumor-associated antigen, B7-H3 actively promotes tumor progression through immune evasion, angiogenesis, oncogenic signaling, metabolic reprogramming, and therapeutic resistance. These diverse biological functions strengthen the rationale for targeting B7-H3 in cancer therapy.

Recent mechanistic studies have substantially expanded our understanding of B7-H3 biology. The mTORC1–B7-H3 axis links PI3K/AKT/mTOR signaling to immune regulation, suggesting that B7-H3 functions as a downstream effector within oncogenic signaling networks (Liu et al., 2023). In parallel, tumor metabolic reprogramming has been directly connected to B7-H3-mediated immune suppression through histone lactylation, providing a mechanistic link between the Warburg effect and the immunosuppressive tumor microenvironment (Ma et al., 2025). Furthermore, the interaction between B7-H3 and inosine-5′-monophosphate dehydrogenase 2 (IMPDH2) identifies a previously unrecognized role in nucleotide metabolism and tumor cell survival (Alhamad et al., 2023). Collectively, these findings position B7-H3 at the intersection of oncogenic signaling, cellular metabolism, epigenetic regulation, and immune modulation, highlighting its potential as a central therapeutic node and providing a strong rationale for combination treatment strategies.

The therapeutic landscape targeting B7-H3 has expanded remarkably and now includes Fc-engineered monoclonal antibodies, antibody–drug conjugates (ADCs), chimeric antigen receptor (CAR) T-cell therapies, bispecific and trispecific immune engagers, and nanobody-based platforms. This diversity reflects the versatility of B7-H3 as a tumor-associated antigen and suggests that no single therapeutic modality is likely to be universally effective across the heterogeneous spectrum of B7-H3-expressing malignancies. Among these approaches, ADCs have advanced most rapidly in clinical development, particularly those employing DNA topoisomerase I inhibitor payloads such as DXd (Yamato et al., 2022).

B7-H3-directed CAR T-cell therapies have generated particularly encouraging early clinical results, especially in central nervous system (CNS) tumors, where conventional immunotherapies are often limited by anatomical and immunological barriers (Ventin et al., 2023). The intraventricular delivery strategy reported by Vitanza et al. represents an important conceptual advance because it bypasses the blood–brain barrier and enables direct access of immune effector cells to intracranial tumors (Yamato et al., 2022). In addition, emerging platforms—including nanobody-based CARs (Li D. et al., 2023) and CAR γδ T cells (Jian et al., 2024)—may overcome several limitations associated with conventional CAR T-cell therapy, including manufacturing complexity, inadequate persistence, and antigen escape.

Beyond these B7-H3-specific approaches, recent advances in next-generation CAR T-cell engineering provide additional strategies that may help overcome major barriers to B7-H3-directed therapy in solid tumors. Emerging approaches include multi-antigen and logic-gated CAR designs to reduce antigen escape, armored CAR T cells engineered to resist immunosuppressive signals, and rational combinations with immune checkpoint inhibitors or other therapies that enhance tumor infiltration. Nanomedicine-enabled strategies may further improve CAR T-cell delivery, trafficking, persistence, and localized activity, whereas metabolic reprogramming may enhance T-cell fitness and reduce exhaustion within the nutrient-depleted tumor microenvironment. In parallel, artificial intelligence and computational approaches may facilitate CAR design optimization, target selection, prediction of effective therapeutic combinations, and biomarker-guided patient stratification (Tan and Zhao, 2024; Lynch et al., 2024). Collectively, integration of advanced CAR engineering with nanomedicine, metabolic modulation, AI-assisted optimization, and rational combination strategies may improve the precision and durability of B7-H3-directed CAR T-cell therapy; however, most of these approaches remain preclinical or early-stage and require further clinical validation (Sharma et al., 2026; Arora et al., 2026).

Despite these advances, several major challenges must be addressed before the full therapeutic potential of B7-H3 can be realized. First, the identity of the physiological receptor for B7-H3 remains unresolved. Without a clearly defined receptor–ligand axis, the molecular mechanisms responsible for its co-inhibitory activity remain incompletely understood, limiting the rational design of targeted therapies (Larkin et al., 2025; Corrigan et al., 2025). Second, each therapeutic platform presents distinct limitations. Although B7-H3-targeted ADCs have demonstrated encouraging clinical activity, low-level B7-H3 expression in normal tissues raises the possibility of on-target/off-tumor toxicity (Mielcarska et al., 2025). Moreover, membrane-permeable cytotoxic payloads may produce bystander killing of adjacent non-malignant cells, potentially narrowing the therapeutic window (Shatzer and Muller, 2026). CAR T-cell therapies have shown promising activity, particularly in solid tumors and CNS malignancies; however, their broader clinical application remains limited by cytokine release syndrome (CRS), immune effector cell-associated neurotoxicity syndrome (ICANS), insufficient persistence, and the highly immunosuppressive tumor microenvironment (Fatahichegeni et al., 2025). Likewise, bispecific and trispecific immune engagers, including trispecific killer engagers (TriKEs), depend on sustained immune-cell activation and may be complicated by excessive cytokine production, immune-cell exhaustion, and reduced efficacy in profoundly immunosuppressed tumors. Third, intratumoral heterogeneity of B7-H3 expression remains a major obstacle across all therapeutic modalities. Variable antigen expression permits the survival and expansion of B7-H3-low or B7-H3-negative tumor cell populations, resulting in antigen escape and therapeutic resistance (Jansen et al., 2025). Consequently, combination strategies targeting multiple tumor antigens or complementary immune pathways will likely be required to achieve durable clinical responses. Finally, the functional duality of B7-H3, initially characterized as a co-stimulatory molecule but now predominantly recognized as co-inhibitory, suggests the existence of context-dependent signaling mechanisms that remain poorly understood (Getu et al., 2023; Mortezaee, 2023; Chen et al., 2020). Importantly, the available evidence does not yet establish whether these apparently opposing activities represent intrinsic properties of a single B7-H3 receptor–signaling axis or arise from interactions with distinct receptors and cellular contexts. Therefore, the classification of B7-H3 as either strictly co-stimulatory or co-inhibitory may be overly simplistic.

One of the most intriguing and unresolved aspects of B7-H3 biology is its apparently paradoxical role in regulating antitumor immunity. Since its identification as a member of the B7 family, B7-H3 has been reported to exhibit both immune-stimulatory and immune-inhibitory activities, leading to longstanding debate regarding its physiological function (Kontos et al., 2021). Initial studies described B7-H3 as a co-stimulatory molecule capable of enhancing CD4^+^ and CD8^+^ T-cell proliferation, interferon-γ (IFN-γ) production, and cytotoxic T-cell activity, suggesting a potential role in promoting antitumor immunity (Chapoval et al., 2001). However, subsequent investigations in both experimental models and human cancers have overwhelmingly supported an immunosuppressive role, demonstrating that B7-H3 inhibits T-cell activation, suppresses natural killer (NK) cell cytotoxicity, promotes regulatory immune cell populations, and facilitates immune evasion within the tumor microenvironment (Mielcarska et al., 2025).

Several factors may account for these seemingly conflicting observations. Foremost, the physiological receptor for B7-H3 has not yet been definitively identified, making it difficult to fully characterize its downstream signaling pathways. It is plausible that B7-H3 interacts with multiple receptors in different cellular contexts, thereby eliciting distinct biological responses. Furthermore, differences in experimental models, species, tissue types, immune-cell composition, and disease stage may contribute to the divergent findings reported in the literature. Early investigations frequently relied on recombinant proteins or overexpression systems under highly controlled experimental conditions, whereas more recent studies have examined endogenous B7-H3 within the complex and chronically immunosuppressive tumor microenvironment (Chapoval et al., 2001).

Accumulating evidence further indicates that B7-H3 possesses important tumor-intrinsic functions that extend well beyond immune checkpoint regulation. B7-H3 has been shown to activate signaling pathways that promote cancer cell proliferation, epithelial–mesenchymal plasticity (EMP), invasion, angiogenesis, metabolic reprogramming, stemness, and resistance to chemotherapy and targeted therapies (Chapoval et al., 2001). These effects have been associated with activation of multiple oncogenic pathways, including PI3K/AKT, JAK/STAT, NF-κB, MAPK, and HIF-1α signaling. Moreover, B7-H3 appears to participate in bidirectional signaling by influencing both immune cells and B7-H3-expressing tumor cells, thereby coordinating reciprocal communication between malignant cells and the surrounding tumor microenvironment (Zhao et al., 2025). Through interactions with macrophages, myeloid-derived suppressor cells (MDSCs), cancer-associated fibroblasts, endothelial cells, and extracellular vesicles, B7-H3 contributes to the establishment of an immunosuppressive, pro-tumorigenic niche that promotes tumor growth, angiogenesis, metastasis, and resistance to therapy (Lu et al., 2024).

The existence of soluble B7-H3 (sB7-H3) further underscores the complexity of B7-H3 biology (Zhao et al., 2026). Elevated circulating concentrations of sB7-H3 have been reported in multiple malignancies and are frequently associated with advanced disease stage and poor clinical outcomes (Dong et al., 2018). Although the mechanisms governing sB7-H3 generation and its biological functions remain incompletely understood, current evidence suggests that soluble B7-H3 may regulate immune responses differently from membrane-bound B7-H3 and may serve as a promising non-invasive biomarker for disease progression and therapeutic response.

Taken together, the available evidence indicates that the biological functions of B7-H3 are highly context dependent. Although early studies suggested an immune-stimulatory role, the overwhelming body of evidence from human cancers supports a predominantly tumor-promoting function characterized by immune suppression, activation of tumor-intrinsic signaling pathways, metabolic adaptation, and extensive remodeling of the tumor microenvironment. Nevertheless, fundamental questions remain regarding the identity of B7-H3 receptors, the molecular mechanisms underlying bidirectional signaling, and the determinants that govern whether B7-H3 exerts immune-stimulatory or immune-inhibitory effects. Addressing these knowledge gaps will be essential for refining our understanding of B7-H3 biology and for guiding the rational development of next-generation B7-H3-targeted immunotherapies.

Beyond oncology, B7-H3 has also emerged as a potential immunoregulatory target in autoimmune and infectious diseases (Xu et al., 2023). In autoimmune disorders, where pathological immune activation drives tissue damage, enhancing or preserving B7-H3-mediated inhibitory signaling may represent a novel immunosuppressive strategy that contrasts with the antagonistic approaches employed in cancer immunotherapy. Conversely, the inducible expression of B7-H3 during infection suggests a broader role in regulating innate and adaptive immune responses (Tigabu, 2024). These contrasting biological contexts underscore the remarkable complexity of B7-H3 biology and emphasize that therapeutic strategies targeting B7-H3 will likely need to be tailored according to disease-specific mechanisms and immune requirements.
